# Impact of radiopharmaceutical therapy (^177^Lu, ^225^Ac) microdistribution in a cancer-associated fibroblasts model

**DOI:** 10.1186/s40658-022-00497-5

**Published:** 2022-09-30

**Authors:** Jonathan Tranel, Stig Palm, Stephen A. Graves, Felix Y. Feng, Thomas A. Hope

**Affiliations:** 1 Department of Radiology and Biomedical Imaging, University of California San Francisco, BH103 1700 4th Street, San Francisco, CA 94158 USA; 2 Department of Medical Radiation Sciences, Institute of Clinical Sciences, Sahlgrenska Academy, University of Gothenburg, Gothenburg, Sweden; 3 Department of Radiology, University of Iowa, Iowa City, IA USA; 4 Department of Radiation Oncology, University of California San Francisco, San Francisco, CA USA; 5 Helen Diller Family Comprehensive Cancer Center, University of California San Francisco, San Francisco, CA USA

**Keywords:** Cancer-associated fibroblasts, Dose voxel kernel, Radiopharmaceutical therapy, Monte Carlo simulation

## Abstract

**Background:**

The aim of this study is to elucidate the difference in absorbed dose (D_abs_) patterns in radiopharmaceutical therapies between alpha emitters (^225^Ac) and beta emitters (^177^Lu) when targeting cancer-associated fibroblasts (CAF) or tumor cells. Five spherical models with 3 mm diameter were created, representing spherical tumor masses that contain tumor clusters, interspersed with CAFs. The mean distance from a tumor cell to the nearest CAF (L_mean_) varied throughout these models from 92 to 1030 µm. D_abs_ calculations were performed while selecting either CAFs or tumor cells as sources, with Convolution/Superposition with ^177^Lu and Monte Carlo simulations (GATE) with ^225^Ac. Analyses were conducted with Dose Volume Histograms and efficacy ratios (ER), which represents the ratio of mean D_abs_ that is deposited in the target volume.

**Results:**

^225^Ac is the most optimal radionuclide when CAFs are both targeted and irradiating themselves, as ERs increase from 1.5 to 3.7 when L_mean_ increases from 92 to 1030 µm. With ^177^Lu, these numbers vary from 1.2 to 2.7. Conversely, when CAFs are sources and tumors are targets with ^225^Ac, ERs decreased from 0.8 to 0.1 when L_mean_ increases from 92 to 1030 µm. With ^177^Lu, these numbers vary from 0.9 to 0.3

**Conclusion:**

When targeting CAFs to irradiate tumors, the efficacy of using ^225^Ac decreases as the average size of the tumor clusters (or L_mean_) increases. In such situations, ^177^Lu will be more effective than ^225^Ac when targeting CAFs due to the longer beta particle range.

**Supplementary Information:**

The online version contains supplementary material available at 10.1186/s40658-022-00497-5.

## Background

Tumors include vascular structures, inflammatory cells, fibroblasts and collagen that together make up the tumor microenvironment or stroma. This stromal support sustains continuous tumor growth as cancer cells reprogram normal fibroblasts into pro-tumorigenic cancer-associated fibroblasts (CAFs), which is a major stroma constituent. CAFs create a niche where tumors are protected from conventional therapies. Consequently, CAFs are becoming a target of interest for diagnosis and prognosis, as depleting them from stroma structure can inhibit cancer growth by disrupting cancer-supportive functions [1].

Fibroblasts activation protein (FAP) is overexpressed on CAFs in many cancers types, such as breast, esophagus, lung, pancreatic, head-neck, colorectal cancers [2]. FAP expression in normal tissues is absent or low, which makes it an appealing target for radiopharmaceutical therapies (RPTs) with antibodies [3], small molecule inhibitors (FAPI) [4] or peptides [5]. As tumor lesions exceeding 1–2 mm in size require the formation of a supporting stroma [6], targeting the stroma can lead to tumor growth suppression as depicted with ^225^Ac-FAPI-04 on xenograft mouse models [4].

Several clinical studies have yielded promising results when targeting FAP. Clinical studies with FAPI-04 [7] and FAPI-46 [8] with ^90^Y demonstrated a significant reduction in patient use of pain medication and a low rate of attributable adverse events on critical organs. In addition, a first clinical feasibility showed encouraging results on advanced adenocarcinomas with ^177^Lu-FAP-2286 peptide [5]. Additionally, studies aiming to improve FAPI time retention can allow a larger flexibility with regard to the choice of the radionuclide [9, 10].

In radiopharmaceutical therapies (RPTs), beta emitters are the most commonly used radionuclides and are employed for the irradiation of large tumors [11]. Targeting of FAP with therapeutic radionuclides is primarily intended to kill neighboring tumor cells, however destruction of CAFs may provide additional benefit. The variability of the spatial distribution of CAFs may play a critical role in the efficacy of CAF-targeted RPT [12]. Although alpha emitters have a higher LET (greater by a factor 500) and shorter ranges (50–100 µm), which may reduce toxicity burden and improve tumor cell killing, the role of ^225^Ac in FAP-targeted RPT remains unknown [4, 13].

To investigate situations when the use of beta or alpha emitters might be optimal when targeting CAFs, we compare their absorbed dose estimates in 3D cellular models with CAFs and tumors intermingling. Modeling was performed with two radioisotopes currently under clinical investigation (^177^Lu and ^225^Ac) representing beta and alpha emitters. To represent the variability of the spatial distribution between CAFs and tumors, several degrees of clustering were modeled.

## Methods

### Analysis of CAFs immunochemistry images

**Fig. 1:**
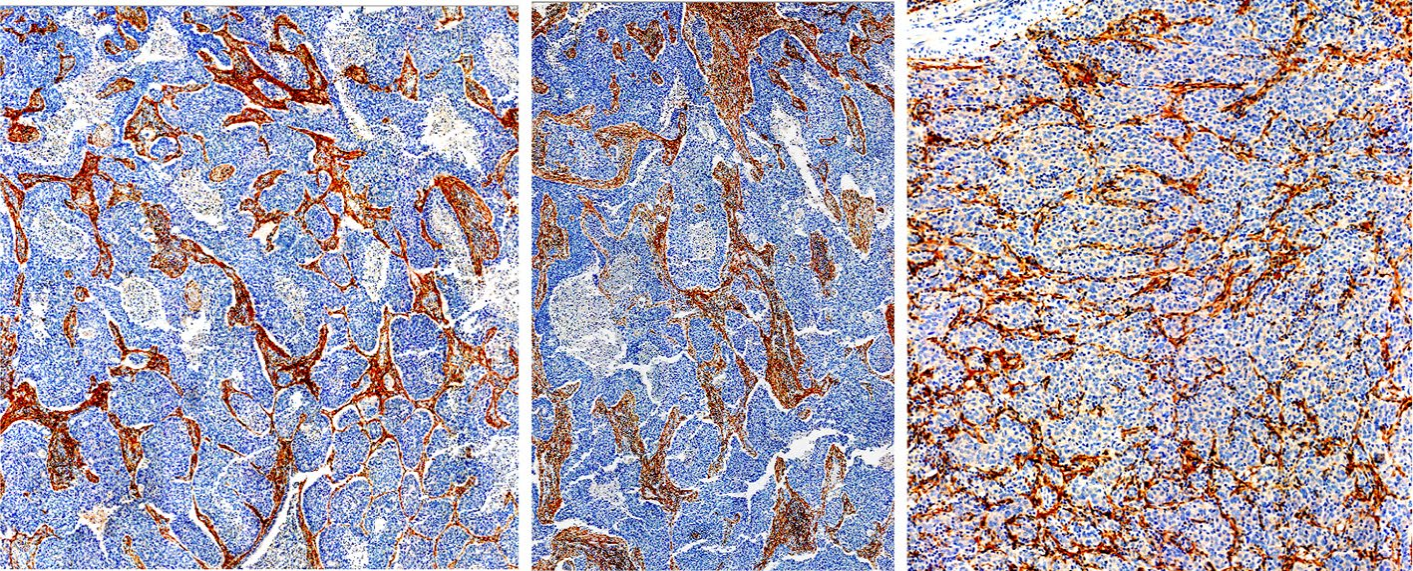
CAFs immunochemistry images of NSCLC (left and middle) and gastric adenocarcinoma (right). Cell nuclei are visible in blue and FAP in red

Figure 1 represents non-small cell lung cancer (NSCLC) and gastric adenocarcinoma. Cell nuclei are stained with the hematoxylin counterstain (blue) and FAP using SP325 FAP antibody (red). To extract information on the spatial distribution of CAF, the pixels of the three CAFs immunochemistry images were first downsampled to 20 µm × 20 µm using 3D Slicer (http://www.slicer.org, 14). Second, CAF and tumors were segmented, considering the non-red areas as tumors, and the distance was calculated between each tumor cell and the nearest CAF (L) along the four cartesian directions using Python 3.7.7. Finally, L distance histograms were plotted and, tumor-to-CAF ratios and L_mean_ were calculated.

### Spherical mass (SM) model and variation of tumor clustering

A 3-mm-diameter spherical model was created as a cellular mass. A voxel sampling of 20 × 20 × 20 μm^3^ was used, corresponding to the approximated dimensions of a cell. Therefore, a continuous distribution was assumed within the SM with a total of 1,767,063 individual voxels. The reference tissue for this model was the liver as it is a common location for metastases [15]. The mass density of 1.05 g.cm^−3^ and the elemental compositions were extracted from the International Commission on Radiological Protection (ICRP 110) adult male computational phantom [16]. Inspired by CAFs immunochemistry images, two types of cells were considered: tumors and the cancer-associated fibroblasts (CAF), with a constant allocation of 75% tumor cells and 25% CAFs within the SM.

**Fig. 2:**
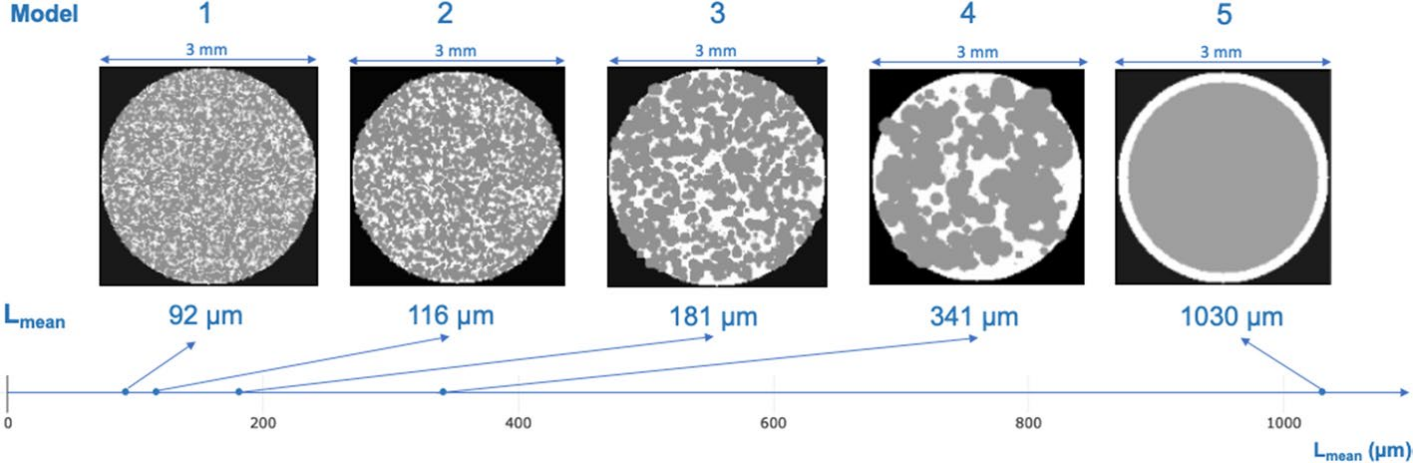
Transversal slice of the five clustering levels of the SM model with tumor cells (gray) surrounded with by CAFs (white) with respective L_mean_

Additionally, tumor cells were gathered into clusters. Five models in total were created where the clusters were adjusted for their size and shape, depicting varying sizes of tumor clusters with interspersed CAFs (Fig. 2). This clustering was quantified by the L_mean_, calculated using the same process as described in the *Analysis of CAF immunochemistry images* but in six cartesian directions as the models are in 3D.

Note that a constant allocation of 75% of tumor cells and 25% of CAFs was maintained within the SM, regardless of the clustering level.

### Dosimetry

Two radioisotopes of interest were selected for the dosimetry part: ^177^Lu for the beta emitters and ^225^Ac for the alpha emitters. ^225^Ac decays with four short-lived alphas emitters (with ^221^Fr, ^217^At and ^213^Bi/^213^Po according to the decay branch), among other minor emissions [17] (Table 1). The energy deposition in the voxels was evaluated for the five SM models with two methods applied to each radioisotope: Convolution/Superposition method with a Dose Voxel Kernel (DVK) for ^177^Lu and a full Monte Carlo modeling of radiation transport (MC) for ^225^Ac.

**Table 1:** Radionuclide properties [11]

Radionuclide	Therapeutic emission	Approximate emission range in tissue (mm)	Radionuclide half-life
^177^Lu	β	0.62	6.6
^225^Ac	α	0.05–0.08	10.0

Tumor cells and CAFs were subsequently designated as sources with equal uptakes, leading to specific analyses on their absorbed dose (D_abs_). As we focused on differences of resulting D_abs_ between tumor geometries, the radioisotopes were modeled to be within the source cells (i.e., the tumors or CAF voxels) and did not redistribute with time (i.e., biological clearance was not considered). In the majority of cancers, FAP is not expressed on tumor cells and therefore CAF and tumors were not considered sources at the same time; although FAP is expressed on sarcoma and mesothelioma tumor cells [8].

To provide approximately realistic activity residence in the tumor, a consistent number of decays were selected for ^177^Lu (10^9^ decays) and ^225^Ac (10^6^ decays). These decays were distributed uniformly among source cells—i.e., 10^9^ decays means that decays per source vary from ~ 755 (when tumor cells are sources) to ~ 2264 (when CAFs are sources). For 10^6^ decays, decays per source vary from ~ 0.8 to 2.3. For ^225^Ac, these variations of decays per source allows some stochastic variability with the spatial uptake distribution when using MC. In the context of alpha-RPT, due to the lower number of decays, the variability of activity per cell is higher with alpha particles compared to beta particles, which cannot be modeled with the Convolution/Superposition method with DVK. With all combinations of source and target, four analyses of D_abs_ were performed. Consistent with the MIRD formalism [18], these combinations of sources and targets can be expressed with the following notation: S(v_Target_ ← v_Source_), leading to the D_abs_ analysis of S(v_Tumors_ ← v_Tumors_), S(v_CAF_ ← v_Tumors_), S(v_Tumors_ ← v_CAF_) and S(v_CAF_ ← v_CAF_). Additional analyses were conducted for the entire SM including CAFs and tumors: S(v_SM_ ← v_CAF_) and S(v_SM_ ← v_Tumors_).

### Convolution/superposition with dose voxel kernels (DVK) for ^177^Lu

For ^177^Lu, the absorbed dose of the radioisotopes was calculated using Convolution/Superposition with a Dose Voxel Kernel (DVK) in Python 3.7.7. The number of primaries used for each SM model was 10^9^ decays. According to the MIRD formalism [18], the total D_abs_ within a voxel is the sum of energy deposition (divided by mass) from all source voxels (1):

1 $${D}_{i,j,k}{(v}_{s})= \sum_{s=0}^{N}\widetilde{A}{(v}_{s}).S({v}_{i, j, k}-{v}_{s})$$

$\widetilde{A}{(v}_{s})$ is the time-integrated activity of the source voxel ${v}_{s}$, directly related with the uptake value, and $S({v}_{i, j, k}-{v}_{s})$ is the absorbed dose in the target voxel ${v}_{i,j,k}$ per decay in the source voxel, which represent the DVK part with a sampling of 20^3^ µm^3^.

DVK methods requires a non-stochastic distribution around the source [19] and therefore was used only for ^177^Lu. As a prerequisite for Convolution/Superposition, ^177^Lu DVK was pre-generated using GATE (Geant4 Application for Tomographic Emission) version 9.0 [20], in the same density and composition as the model, namely the liver (1.05 g.cm^−3^), extracted from the ICRP 110 [16]. The maximal beta emission range of ^177^Lu is 1.8 mm, thus, a specific filter size of 201^3^ voxels (2 mm range) was selected according to the radionuclide physical properties, so that the filter size encompasses more than 99% of the respective total energy deposition. The numbers of decays used for DVK generation for ^177^Lu were 10^7^ which resulted in a relative standard deviation for the absorbed dose at the DVK source voxel of less than 0.04% (5% at 0.4 mm from the source). More detailed parameters for the DVK generation are common with the direct MC simulation, available in Monte Carlo simulation (MC) for ^225^Ac.

### Monte Carlo simulation (MC) for ^225^Ac

MC simulations were performed for ^225^Ac using GATE [20] version 9.0 (release date: 03–2020) using 10^6^ particles for each SM model. The *Livermore* physics model was selected which considers all atomic shells and has the best agreement with validation studies performed to low energies down to 10 eV [21]. In the simulations, the step size limit or range cut-off parameter was arbitrary chosen to 1/20th of the voxel size, i.e., 1 μm. The GATE Radioactive Decay Module was enabled to ensure the full decay chain and its associated emissions were simulated [21], and the Mersenne-Twister engine was selected. The entire energy spectra from parents and all daughters were considered in the Monte Carlo models of the deposited energy resulting from the radiation emitted from these radioisotopes. The model was embedded in a *world* size of 10^3^ cm^3^.

### Analysis of the SM models

#### Absorbed dose (D_abs_) maps and statistics

D_abs_ maps were created using 3D Slicer in 3D. Second, the percentage of target cells that received ≥ 10% of the maximum D_abs_ were calculated for both sources across the five tumor models. Third, the mean D_abs_ within the SM, including both CAFs and tumor cells, was calculated, e.g., S(v_SM_ ← v_CAF_) and S(v_SM_ ← v_Tumors_).

#### Dose volume histograms (DVH)

DVHs were calculated for S(v_Tumors_ ← v_Tumors_), S(V_CAF_ ← V_CAF_), S(v_Tumors_ ← v_CAF_) and S(v_CAF_ ← v_Tumors_) using Python 3.7.7. for both ^177^Lu and ^225^Ac using CAFs and tumor as targets and sources.

#### Efficacy ratios (ER)

Ratios of mean D_abs_ were calculated for S(v_Tumors_ ← v_CAF_) and S(V_CAF_ ← V_CAF_) relative to S(v_SM_ ← v_CAF_), and for S(v_Tumors_ ← v_Tumors_) and S(v_CAF_ ← v_Tumors_) relative to S(v_SM_ ← v_Tumors_) termed as efficacy ratios (ERs). The ER represents the fraction of mean D_abs_ measured on SPECT or PET imaging (when appropriate quantification is feasible) that is deposited in the target volume. The main difference with the MIRD 21 absorbed fraction [22] is that ER do not consider the energy escaped out of the SM. An ER of 1 means that the target volume received an D_abs_ equal to the total of the D_abs_ of the SM.

## Results

### Analysis of CAFs immunochemistry images

**Fig. 3:**
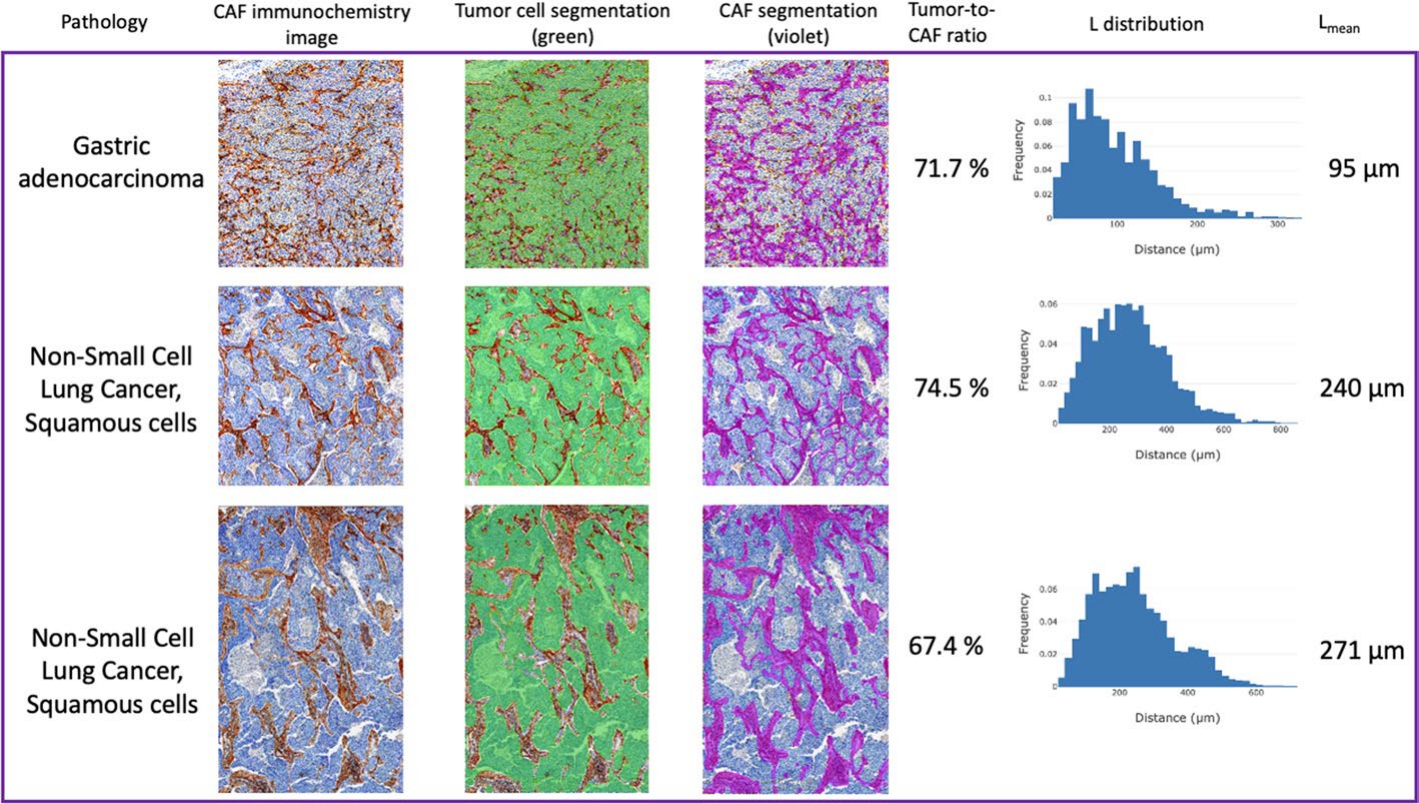
Three images of CAF immunochemistry with associated CAF and tumor segmentation, tumor ratio, nearest CAF for each tumor distance (L) distribution and L_mean_

Figure 3 displays the three CAFs immunochemistry images with their respective CAFs and tumors segmentations, tumor ratios, L distributions and L_mean_. Note that the segmentations and subsequent calculations were performed with a 20 µm × 20 µm undersampling.

The three CAFs immunochemistry images depict similar tumor-to-CAF ratios, with an average of 71.2% tumor cells, and an L_mean_ ranging from 95 to 271 µm (Fig. 3). Note that the L_mean_ of the five SM models covers the L_mean_ of the CAFs immunochemistry images (92 to 1030 µm, see Additional file 1: Fig. S1).

### Analysis of the SM model

#### Absorbed dose (D_abs_) maps and statistics

**Fig. 4:**
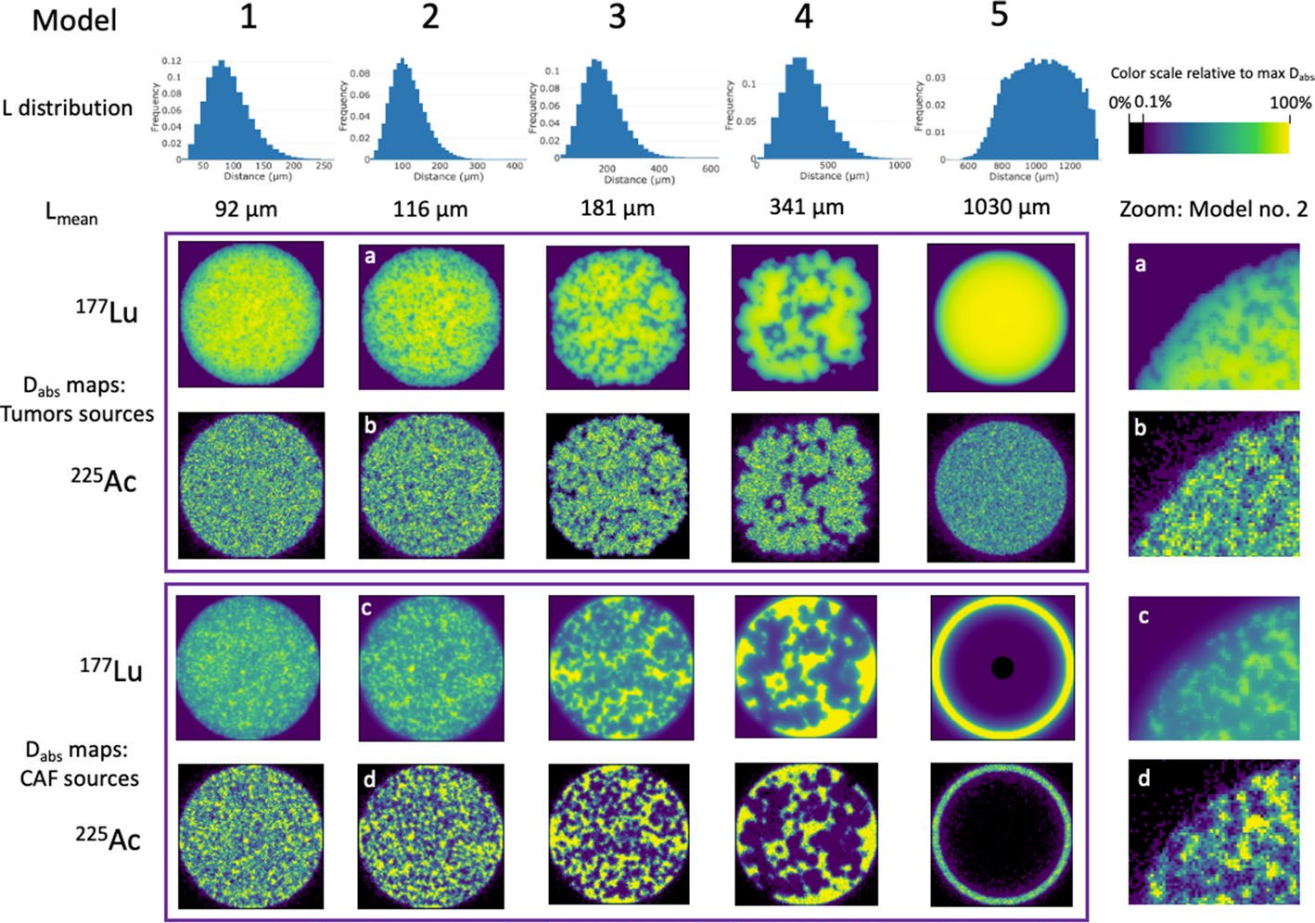
Representative slices of the five models with their associated L_mean_ (left column: grey are tumor cells, white are CAFs) and associated D_abs_ maps with tumor cells and CAFs as sources for ^177^Lu and ^225^Ac. Magnifications of the upper left corner of model 2 are provided to show the differences in stochastic noise between the radionuclides (right column)

D_abs_ maps were analyzed for both radioisotopes in two cases: with either CAFs or tumors as sources (Fig. 4).

**Fig. 5:**
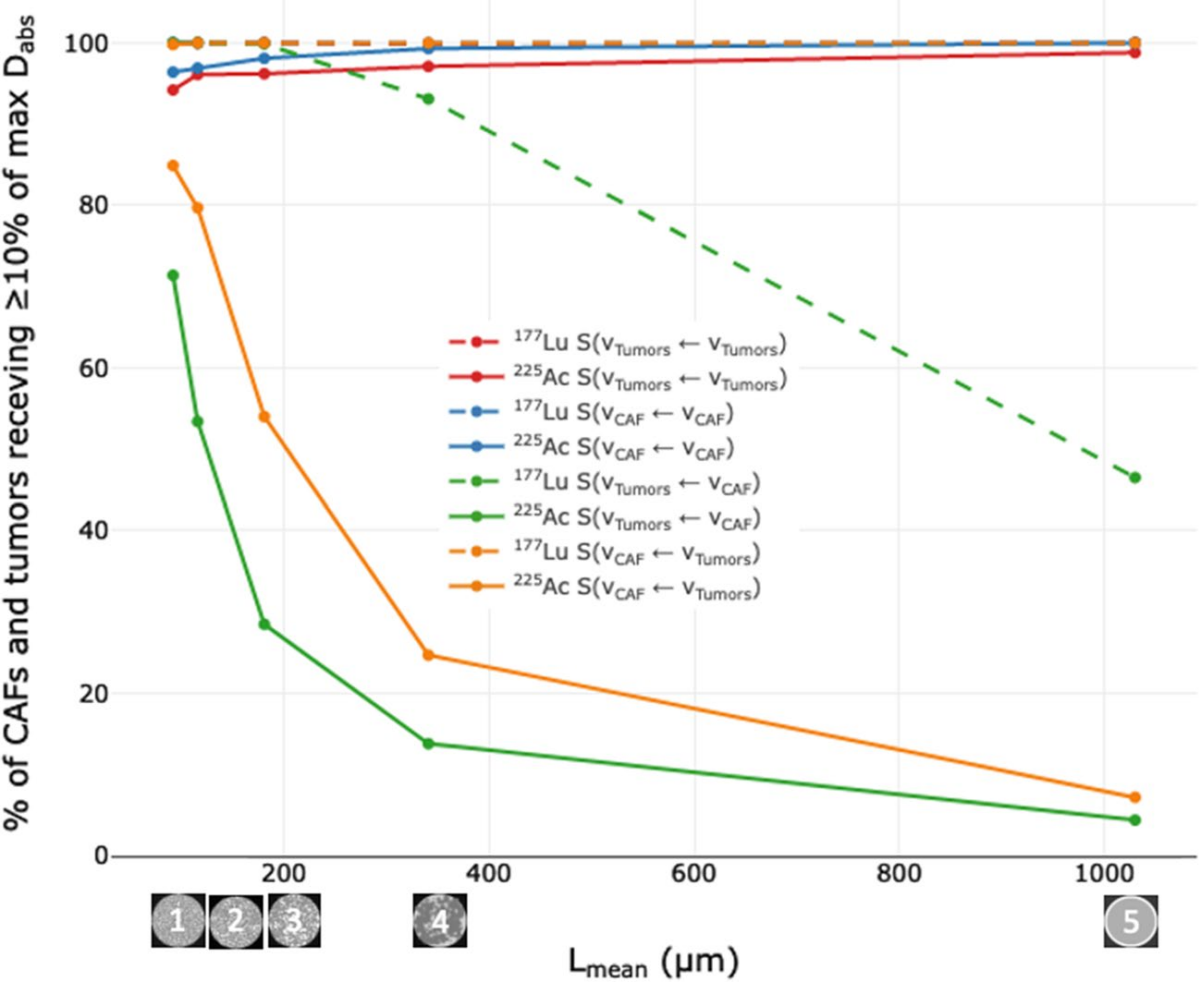
Percentage of CAFs and tumors receiving ≥ 10% of the maximum D_abs_ within the SM across the five models using either tumor cells or CAFs as sources or targets for ^177^Lu and ^225^Ac. Values for ^177^Lu S(v_Tumors_ ← v_Tumors_), S(V_CAF_ ← V_CAF_) and S(V_CAF_ ← V_Tumors_) are superposed

The D_abs_ maps demonstrate that ^177^Lu is associated with a more homogeneous appearing D_abs_ than ^225^Ac. This is confirmed by the high percentages of CAFs and tumors cells receiving D_abs_ ≥ 10% of the maximum D_abs_ (Fig. 5, Additional file 1: Table S1). Conversely, the use of ^225^Ac shows a more heterogeneous D_abs_, as demonstrated by the low percentage of CAFs and tumors cells receiving ≥ 10% of the maximum D_abs_.

The lowest percentage of CAFs and tumor cells receiving ≥ 10% of the maximum D_abs_ is seen with ^225^Ac for the model 5. In this model, when CAFs are sources and tumors are targets (i.e., S(V_Tumors_ ← V_CAF_) in solid green), 4.4% of the tumor cells receive ≥ 10% of the maximum D_abs_. Additionally, the mean D_abs_ within the SM is minimally impacted by clustering for both ^177^Lu and ^225^Ac (Additional file 1: Fig. S2).

#### Dose volume histograms (DVH)

**Fig. 6:**
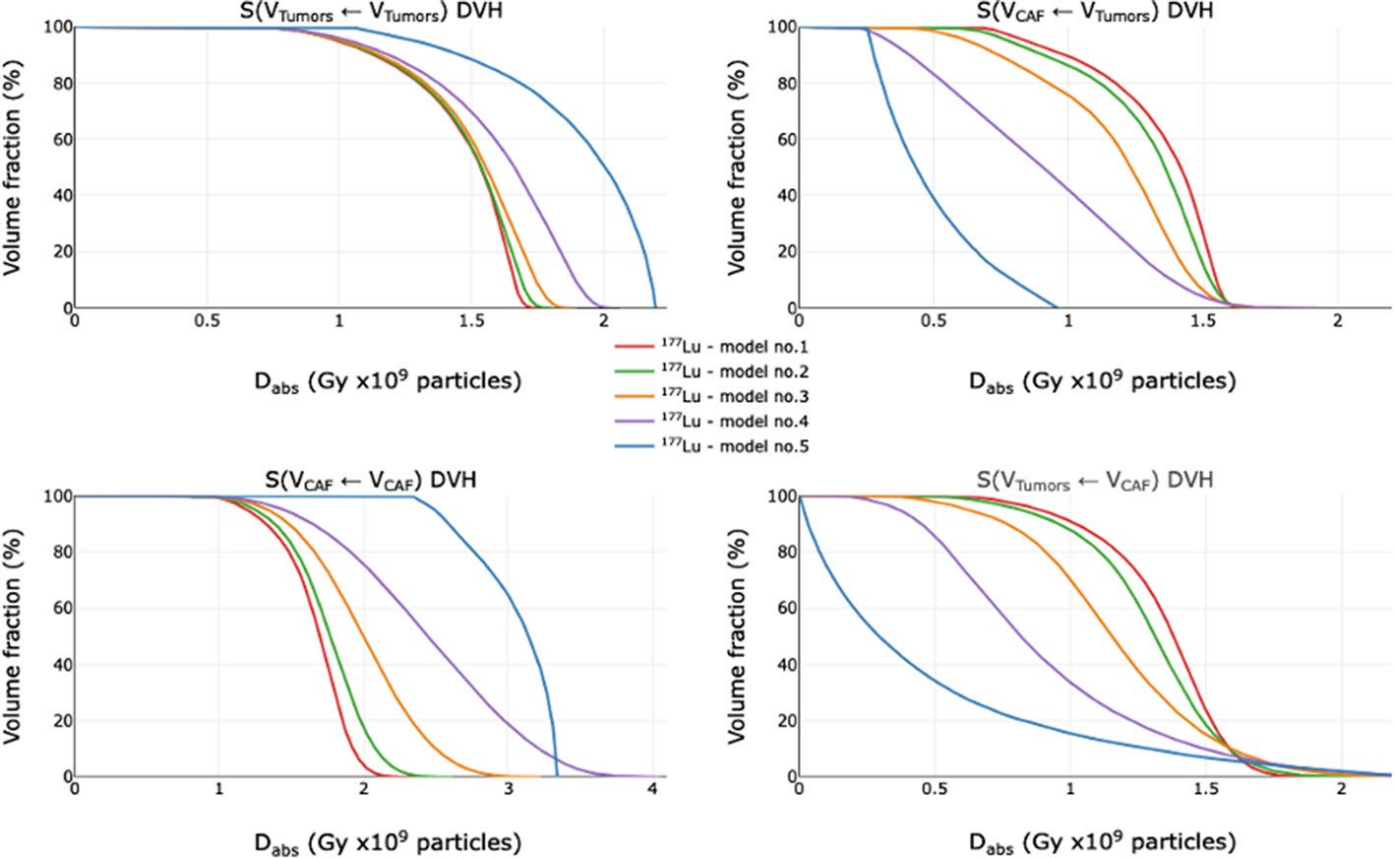
^177^Lu Dose Volume Histograms of the five models for S(v_Tumors_ ← v_Tumors_), S(v_CAF_ ← v_Tumors_), S(v_Tumors_ ← v_CAF_) and S(v_CAF_ ← v_CAF_)

**Fig. 7:**
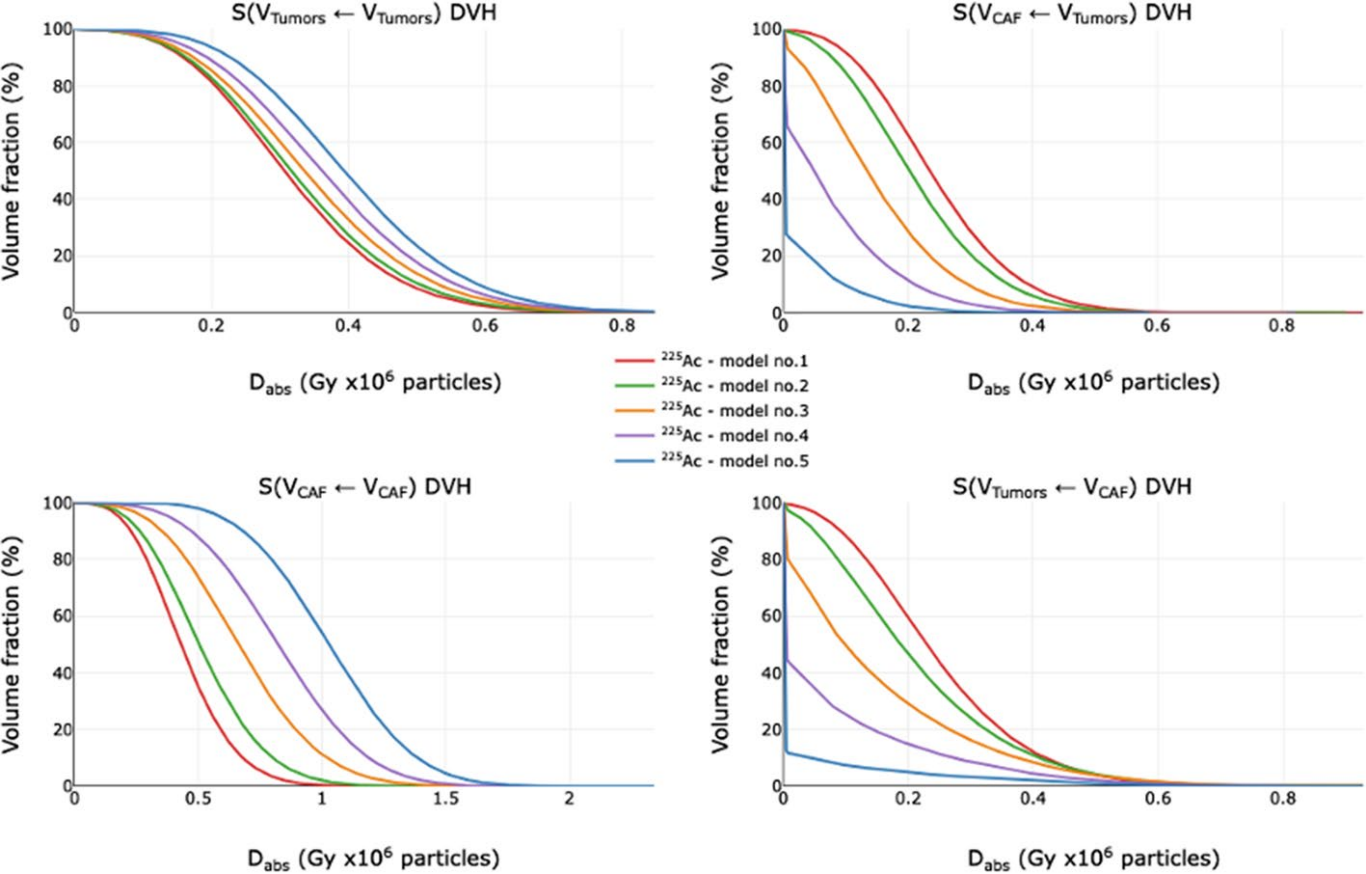
^225^Ac Dose Volume Histograms of the five models for S(v_Tumors_ ← v_Tumors_), S(v_CAF_ ← v_Tumors_), S(v_Tumors_ ← v_CAF_) and S(v_CAF_ ← v_CAF_)

DVHs were plotted in Fig. 6 for ^177^Lu and in Fig. 7 for ^225^Ac for the four combinations of targets and sources: S(v_Tumors_ ← v_Tumors_), S(v_CAF_ ← v_Tumors_), S(v_Tumors_ ← v_CAF_) and S(v_CAF_ ← v_CAF_).

For all the models, ^177^Lu and ^225^Ac are the most effective when targets and sources are identical (S(v_Tumors_ ← v_Tumors_) and S(v_CAF_ ← v_CAF_) in left columns). This trend is most critical for the model 5, where L_mean_ is the highest. In addition, the DVH slopes of ^177^Lu are steeper than those of ^225^Ac, due to a more homogeneous D_abs_, as observed in 3.2.1. In the setting where sources and targets are different (S(v_CAF_ ← v_Tumors_) and S(v_Tumors_ ← v_CAF_) in right columns), the opposite is true. The shoulders of the DVH slopes for ^225^Ac become sharper when going from model 1 to 5. With ^225^Ac, when the targets and sources are different, a large percentage of the targets receive negligible doses as shown by extrapolated shoulder y-intercepts of less than 100%. For example, with model 5, 73% of CAFs for S(v_CAF_ ← v_Tumors_) and 88% of tumors for S(v_Tumors_ ← v_CAF_) receive nominally negligible dose. With model 1, this effect is minimized with only 2% of CAFs for S(v_CAF_ ← v_Tumors_) and 0% of tumors for S(v_Tumors_ ← v_CAF_) receiving negligible dose. Additionally, the mean D_abs_ within the SM is minimally impacted by clustering for both ^177^Lu and ^225^Ac (Additional file 1: Fig. S2).

#### Efficacy ratios (ER)

**Fig. 8:**
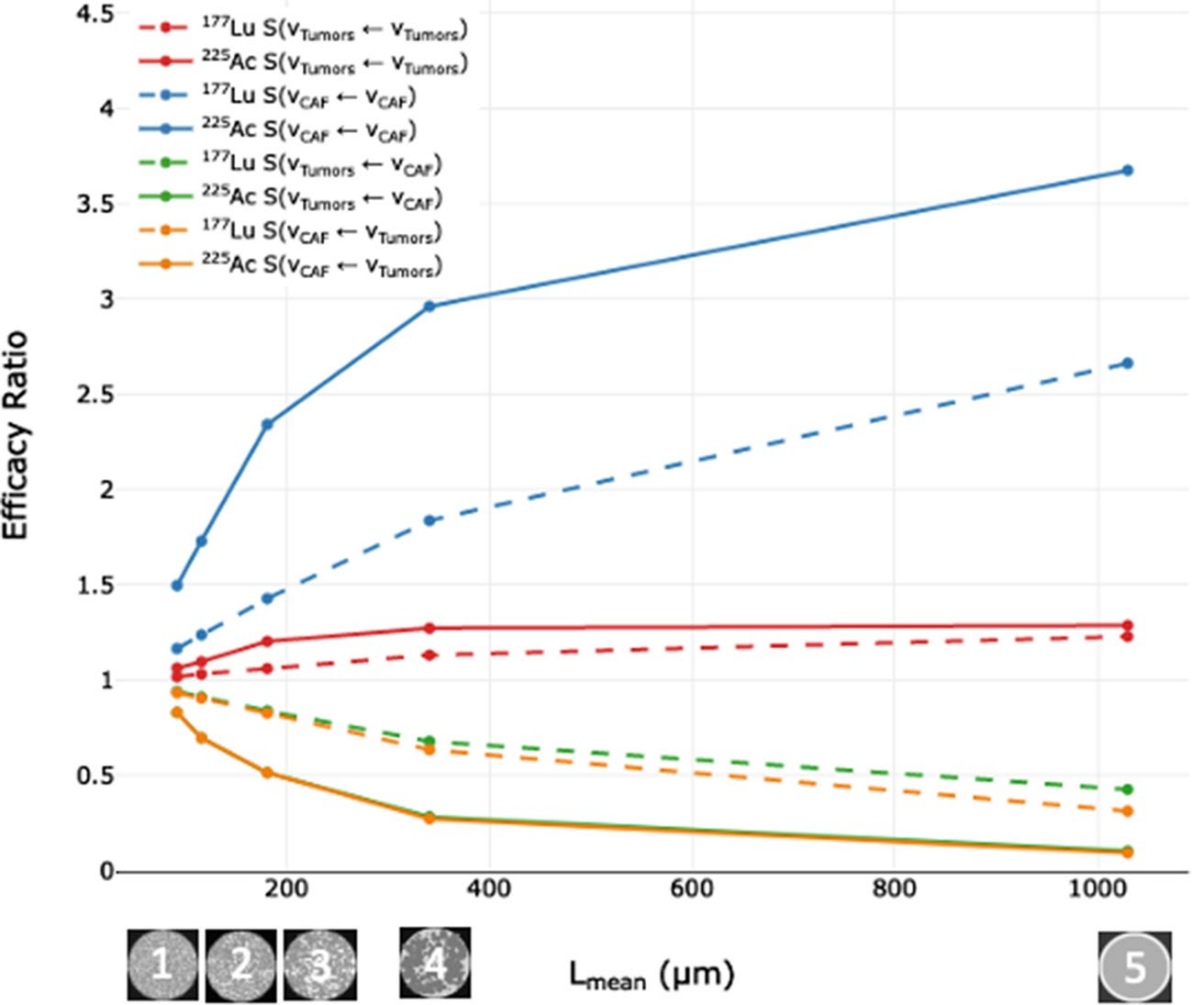
Efficacy ratios (ERs) of the five models using either tumors and CAFs as sources or targets for ^177^Lu and ^225^Ac. Values for ^225^Ac S(v_Tumors_ ← v_CAF_) and (v_CAF_ ← v_Tumors_) are superposed

ERs are plotted in Fig. 8 for ^177^Lu and ^225^Ac for S(v_CAF_ ← v_Tumors_), S(v_Tumors_ ← v_CAF_) and S(v_CAF_ ← v_CAF_) and S(v_Tumors_ ← v_Tumors_).

When L_mean_ increases (from 92 µm to 1030 µm), the most optimal combination is obtained with ^225^Ac and S(v_CAF_ ← v_CAF_), with the ER reaching 3.7 in model 5. Conversely, the ER of ^225^Ac with S(v_CAF_ ← v_Tumors_) is the lowest at 0.1. Overall, ^225^Ac is the most impacted by changes in L_mean_, with the ER increasing 2.2 (+ 147%) for S(v_CAF_ ← v_CAF_), and decreasing 0.73 (−87%) for S(v_Tumors_ ← v_CAF_) and 0.74 (−88%) for S(v_CAF_ ← v_Tumors_) when going from model 1 to 5. The impact of L_mean_ is more muted for ^177^Lu, with the ER increasing 1.5 (+ 125%) with S(v_CAF_ ← v_CAF_) and decreasing 0.6 (−66%) with S(v_CAF_ ← v_Tumors_) when going from model 1 to 5.

## Discussion

In this work, we have modeled a tumor comprised of a fixed ratio of tumor cells and CAFs, varying the cluster size of the tumor cells. We have shown that the use of alpha emitters results in a significant fraction of the target mass that receives negligible absorbed dose, which becomes more pronounced as clustering increases. Impact of clustering on target absorbed dose with beta particles is more muted than with alpha particles when the targets and sources are not the same, such as what would be the case with FAP-targeted RPT.

The reason for this effect on cluster size for alpha particles is due its short range (~ 60 µm). Therefore, the effect of crossfire decreases when the mean distance between tumors and CAF (L_mean_) increases. In contrast, due to their larger range (~ 0.6 mm for ^177^Lu [11]), beta emitters benefit from crossfire irradiation as the clustering size increases, making beta particles more effective in larger clusters compared to alpha particles. The benefit of ^177^Lu is limited when the cluster size is larger than ~ 600–700 µm, which correspond to its maximal range in tissues.

It is interesting to note that ^225^Ac ERs do not demonstrate a significant advantage over ^177^Lu ERs when tumors are both sources and target or S(v_Tumors_ ← v_Tumors_). This is due to the high tumor cellular ratio (75% of the volume) which increases the probability of crossfire effect for ^177^Lu. An inverted ratio would favor the use of ^225^Ac over ^177^Lu.

Understanding CAFs spatial distributions across types may help to personalize RPTs in regards to the choice of appropriate radionuclide [1]. As an example, sarcoma and mesothelioma express FAPs on tumor cells [8], and therefore the impact of clustering may be muted. In most tumors, FAP is not expressed on tumor cells [23], and in these cases, consideration of clustering impacts the choice of radionuclide used.

The overall deposited dose (mean D_abs_) in the SM models was relatively consistent independent of clustering. This indicates that D_abs_ discrepancies due to the selection of CAF or tumors as sources, and various L_mean_ are not discernable when measuring the mean D_abs_ at the macroscopic scale, as performed in nuclear medicine with SPECT or PET [24]. This is consistent with prior work, which has shown that measuring to the mean D_abs_ at the organ level may be inaccurate for quantifying biological effects, especially for alpha emitters [25, 26]. For this reason, one must be careful when applying the promising imaging results of FAPI PET to predict subsequent efficacy to FAP-targeted RPT [2].

Our dosimetry results are consistent with the pancreas mouse model (PANC-1), using ^177^Lu and ^225^Ac with FAPI-46 [13]. The authors observed that ^177^Lu effects were marginally superior to ^225^Ac. It was assumed that these effects were due to a better D_abs_ homogeneity throughout the tumor mass, whereas ^225^Ac irradiation were locally limited. These observations are consistent with a L_mean_ greater than 100 µm although these measures were not reported [13].

Limitations of this study are primarily the lack of in vitro correlates to our modeling. Additionally, further studies on the spatial distribution of CAFs and tumor cells are required to better elucidate clustering in vivo, and to better understand the relative benefit of alpha or beta emitters. This work could also be extended to comparison of ^90^Y against ^177^Lu (particularly when L_mean_ might exceed 500 µm), motivated by the interesting results of a ^90^Y-FAPI-46 feasibility study [8]. Additionally, tumor mass radius greater than 3 mm, various tumor-to-CAF ratios or heterogeneous uptake distributions could be considered in our model, as CAFs and associated fibrosis can hamper the accessibility of RPTs within the tumoral mass [1].

In order to focus on the radiation properties of the isotopes, the complex biological reality was simplified. For example, CAFs and tumor cells were modeled as cubes and the entire SM volume was considered as sensitive and used for calculation of absorbed dose. Re-distribution of the parent or the ^225^Ac daughters were not simulated. Various developments for retaining ^225^Ac and its daughter radioisotopes are ongoing and might strength this approximation in the future. These techniques include for example the use of containing polymersomes containing nanoparticles, which revealed a ^213^Bi retention of at least 69% and a much-decreased renal uptake of free ^213^Bi compared to no retention strategies at all [27, 28].

Also, clearance was not considered, although several authors reported that the tumor retention time were particularly short for FAPI-02 and FAPI-04 [4, 9], which can mitigate the selection of ^225^Ac and ^177^Lu, beyond the criterion of L_mean_. If biological half-life were modeled, shorter half-life radionuclides would have potential benefit such as ^211^At. The alternative to shorter half-life radionuclides is to improve the retention of the radioligand as shown in recent promising results for FAPI-21, FAPI-46 [9], and FAP-2286, even if their performance remain lower than ^177^Lu-PSMA, ^177^Lu-DOTATOC, or ^177^Lu-DOTATATE [5].

Another limitation is that this work did not model the difference in labelling rates between ^177^Lu and ^225^Ac. Labelling rates for ^177^Lu are around 20 FAP-ligand molecules per ^177^Lu atom [29], versus one ^225^Ac atom per million molecules [4]. This much lower labelling efficiency may result in target saturation, limiting CAF uptake of alpha labeled FAP-radioligands. Additionally, we based our efficacy criterion on the irradiation of the target cells in the spherical mass, ignoring bystander effects. However, DNA damage, cell death, apoptosis and cell transformation occurs even in non-irradiated cells [30].

Finally, this work is based on a spherical model which cannot represent the large heterogeneity of the CAF and tumor existing structures. The 75%/25% tumor-to-CAF ratio is not representative of all tumors but was selected as an example case, supported by the data of the three CAFs immunochemistry. Future work can explore more various tumor-to-CAF ratio, and heterogeneous uptakes which are specific of other pathologies. Furthermore, the blood vessels, immune cells or acellular components were not modeled, for simplification. However, we believe that our results are still informative as the lack of other components does not impact the specific expression of FAP either by CAF or tumor cells.

## Conclusion

Our work demonstrated that changes in tumor cell clustering may impact the efficacy of FAP-targeted RPTs, particularly with ^225^Ac due to the short radiation range. Measured absorbed dose using SPECT or PET will overestimate the relative benefit of alpha particles compared to beta particles when targeting CAFs. The longer radiation range of ^177^Lu helps mitigate the effect of cluster size. An improved understanding of tumor microenvironment distribution (CAFs and tumor cells) may help to optimize RPTs with respect to the choice of radionuclide (alpha or beta emitting agents) and the source/target combination (tumors and/or CAFs).

## Supplementary Information

**Additional file 1:** **Fig. S1**. Lmean rank of the five SM models and CAFs immunochemistry. **Table S1**. Percentage of CAFs and tumors receiving ≥10 % of the maximum Dabs within the SM model of the five models using either tumors and CAFs as sources for 177Lu and 225Ac. **Fig. S2**. Mean Dabs within the SM model of the five models using either tumors (brown) and CAFs (violet) as sources for 177Lu (dashed) and 225Ac (solid).

## Data Availability

The datasets used and/or analyzed during the current study are available from the corresponding author on reasonable request.

## Abbreviations

D_abs_: Absorbed dose
RPT: Radiopharmaceutical therapy
CAF: Cancer-associated fibroblast
DVH: Dose volume histogram
ER: Efficacy ratio
FAP: Fibroblast activation protein
FAPI: Fibroblast activation protein inhibitor
NSCLC: Non-small cell lung cancer
L: Distance between each tumor cell and the nearest CAF
L_mean_: Mean distance between each tumor cell and the nearest CAF
SM: Spherical mass
ICRP: International Commission on Radiological Protection
MC: Monte Carlo
MIRD: Medical Internal Radiation Committee
GATE: Geant4 Application for Tomographic Emission
SPECT: Single photon emission computed tomography
PET: Positron emission tomography
PSMA: Prostate-specific metastasis antigen
DOTATOC: DOTA0-D-Phe1-Tyr3-octreotide
