# Supplementary material for: Impact of radiopharmaceutical therapy (177Lu, 225Ac) microdistribution in a cancer-associated fibroblasts model
Source: EJNMMI Phys. 2022 Sep 30;9:67. doi: 10.1186/s40658-022-00497-5 (PMC9525486; doi:10.1186/s40658-022-00497-5)
Supplement: Supplementary file 1 — Additional file 1: Fig. S1. Lmean rank of the five SM models and CAFs immunochemistry. Table S1. Percentage of CAFs and tumors receiving ≥10 % of the maximum Dabs within the SM model of the five models using either tumors and CAFs as sources for 177Lu and 225Ac. Fig. S2. Mean Dabs within the SM model of the five models using either tumors (brown) and CAFs (violet) as sources for 177Lu (dashed) and 225Ac (solid). [file 40658_2022_497_MOESM1_ESM.docx]

**Additional file 1**


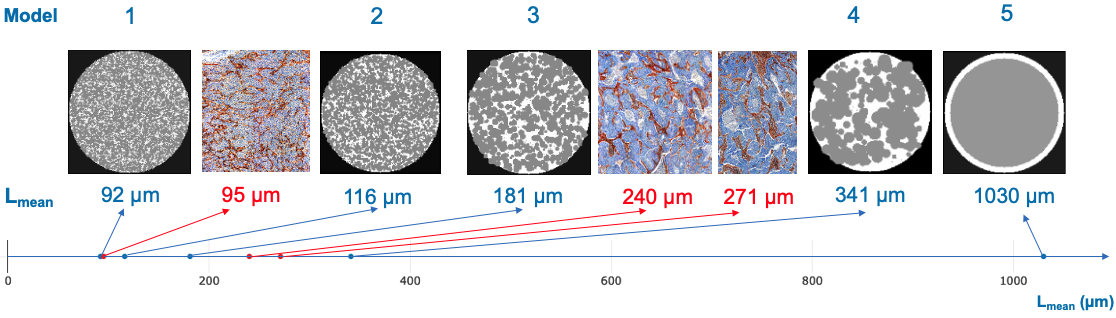


Additional file 1: Fig. S1 – L_mean_ rank of the five SM models and CAFs immunochemistry.

|  | | CAF sources | | | | Tumor sources | | | |
| --- | --- | --- | --- | --- | --- | --- | --- | --- | --- |
|  |  | ^177^Lu | | ^225^Ac | | ^177^Lu | | ^225^Ac | |
| Model | L_mean_ | CAF | Tumors | CAF | Tumors | CAF | Tumors | CAF | Tumors |
| 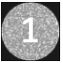 | 92 µm | 100% | 100% | 96.4% | 71.4% | 99.8% | 100% | 84.9% | 94.2% |
| 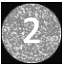 | 116 µm | 100% | 100% | 96.9% | 53.4% | 100% | 100% | 79.7% | 96.1% |
| 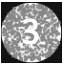 | 181 µm | 100% | 99.9% | 98.1% | 28.5% | 100% | 100% | 54.0% | 96.2% |
| 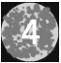 | 341 µm | 100% | 93.1% | 99.3% | 13.8% | 100% | 99.8% | 24.7% | 97.1% |
| 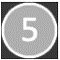 | 1030 µm | 100% | 46.5% | 100% | 4.4% | 100% | 100% | 7.2% | 98.8% |

Additional file 1: Table S1 – Percentage of CAFs and tumors receiving ≥10 % of the maximum D_abs_ within the SM model of the five models using either tumors and CAFs as sources for ^177^Lu and ^225^Ac.


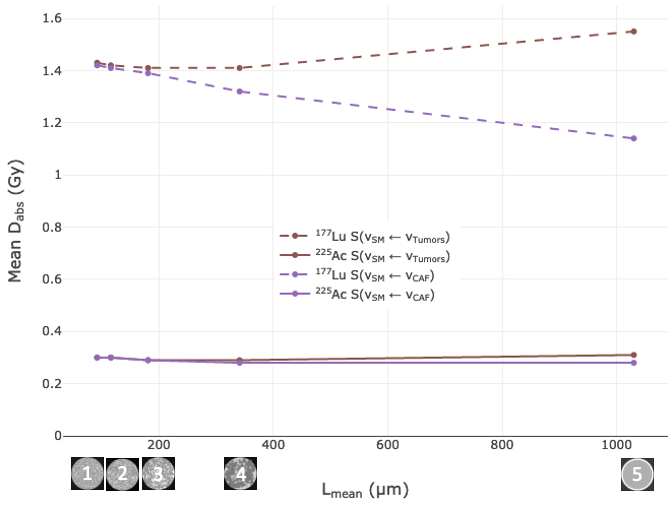


Additional file 1: Fig. S2 – Mean D_abs_ within the SM model of the five models using either tumors (brown) and CAFs (violet) as sources for ^177^Lu (dashed) and ^225^Ac (solid).
